# Extended adjuvant temozolomide in newly diagnosed glioblastoma: A single-center retrospective study

**DOI:** 10.3389/fonc.2022.1000501

**Published:** 2022-11-22

**Authors:** Jie Chen, Tingting Wang, Wanming Liu, Hui Qiu, Nie Zhang, Xueting Chen, Xin Ding, Longzhen Zhang

**Affiliations:** ^1^ Department of Radiation Oncology, Affiliated Hospital of Xuzhou Medical University, Xuzhou, China; ^2^ Cancer Center, Xuzhou Medical University, Xuzhou, China; ^3^ First Clinical College, Xuzhou Medical University, Xuzhou, China

**Keywords:** glioblastoma, extended adjuvant temozolomide, MGMT methylation, IDH1 mutation, prognosis

## Abstract

**Objective:**

To investigate whether extending adjuvant temozolomide (TMZ) improved the prognosis of newly diagnosed glioblastoma (GBM) patients with different mutation statuses of O^6^-methylguanine DNA methyltransferase (MGMT), isocitrate dehydrogenase 1 (IDH1), p53 and different expression level of Ki67.

**Methods:**

This study was a retrospective cohort study that postoperative patients with newly diagnosed GBM who did not progress after receiving radiotherapy with concomitant and 6 cycles of adjuvant TMZ were enrolled in control group, and those received more than 6 cycles of adjuvant TMZ were incorporated in extended group. Patients were stratified by MGMT expression, IDH1 mutation, p53 mutation and expression level of Ki67. The primary endpoints were overall survival (OS) and progression-free survival (PFS).

**Result:**

A total of 93 postoperative patients with newly diagnosed GBM were included in this study, 40 and 53 cases were included in control group and extended group, respectively. On the whole, extended adjuvant TMZ chemotherapy significantly prolonged OS and PFS of patients with newly diagnosed GBM [median OS (mOS): 29.00 months *vs.* 16.70 months, *P* < 0.001; median PFS (mPFS): 13.80 months *vs.* 9.60 months, *P* = 0.002]. The results of subgroup analysis showed that patients with methylated MGMT in extended group had significantly longer OS and PFS than those in control group; patients with IDH1 mutation benefited more from extended adjuvant TMZ chemotherapy than those with wild-type IDH1; there was no significant difference in the effect of extended TMZ chemotherapy on OS between GBM patients with wild-type p53 and those with mutant p53; compared with GBM patients with lower expression of Ki67, extended adjuvant TMZ treatment dramatically improved the OS and PFS of those with higher expression of Ki67.

**Conclusion:**

The therapeutic schedule of extended adjuvant TMZ significantly prolonged OS and PFS of patients with newly diagnosed GBM regardless of p53 mutation status, and patients with different MGMT methylation, IDH1 mutation and Ki67 expression level benefited differently from extended adjuvant TMZ chemotherapy.

## Introduction

Glioblastoma (GBM) is a highly fatal and the most common primary malignant intracranial tumor in adults with limited treatment options (1), the standard-of-care therapy for newly diagnosed GBM is surgical resection of tumor to the extent safely feasible, followed by concurrent radiochemotherapy with temozolomide (TMZ) and 6 cycles of adjuvant TMZ chemotherapy——STUPP regimen (2, 3), yet the therapeutic effect of this standard regimen is not particularly satisfactory, although it extends patients’ median overall survival (mOS) from 12.1 months to 14.6 months, the two-year and five-year survival rates remain low (26.5% and 9.8%, respectively) (3).

In an effort to further prolong the survival of GBM patients and improve their prognosis, experts in GBM have attempted to put forth new strategies on the basis of STUPP regimen. Compared with other antineoplastic drugs previously used for patients with newly diagnosed GBM, TMZ was significantly better tolerated and had fewer side effects, which made it became an irreplaceable option for these population (2). Hence, studies on adjusting the duration and dose of TMZ have been conducted, and some clinicians have extended the number of treatment cycles to 12 or even more in nonprogressive GBM patients, despite lack of sufficient data (4). Although some studies showed that extended TMZ therapy was well tolerated and contributed to a significant increase in survival time in patients with newly diagnosed GBM (5–7), some negative results were reported one after another, for example, Gilbert MR et al. demonstrated that there was no significant difference between standard TMZ treatment and dose-dense TMZ for 6 to 12 cycles in mOS or median progression-free survival (mPFS), regardless of methylation status (8); the study of Balana C et al. suggested that no apparent clinical benefit of continuing TMZ treatment for more than 6 cycles was appeared (9); the results of clinical trial GEINO 14-01 indicated that 12 cycles of TMZ treatment did not confer additional clinical benefits for OS and PFS, even in GBM patients with O (6)-methylguanine DNA methyltransferase (MGMT) promoter methylation (10). Therefore, the optimal duration of TMZ maintenance therapy remains a matter of debate and deserves further investigation.

In addition, there is no studies investigated the effect of isocitrate dehydrogenase 1 (IDH1) mutation, p53 mutation and Ki67 expression level on the therapeutic effect of extended TMZ chemotherapy. Consequently, we set out to accumulated more evidence to make robust recommendations for the clinical use of extended adjuvant TMZ chemotherapy and to assist clinicians in identifying specific population with newly diagnosed GBM that would benefit from extended TMZ treatment.

## Patients and methods

### Study design

**Figure 1:**
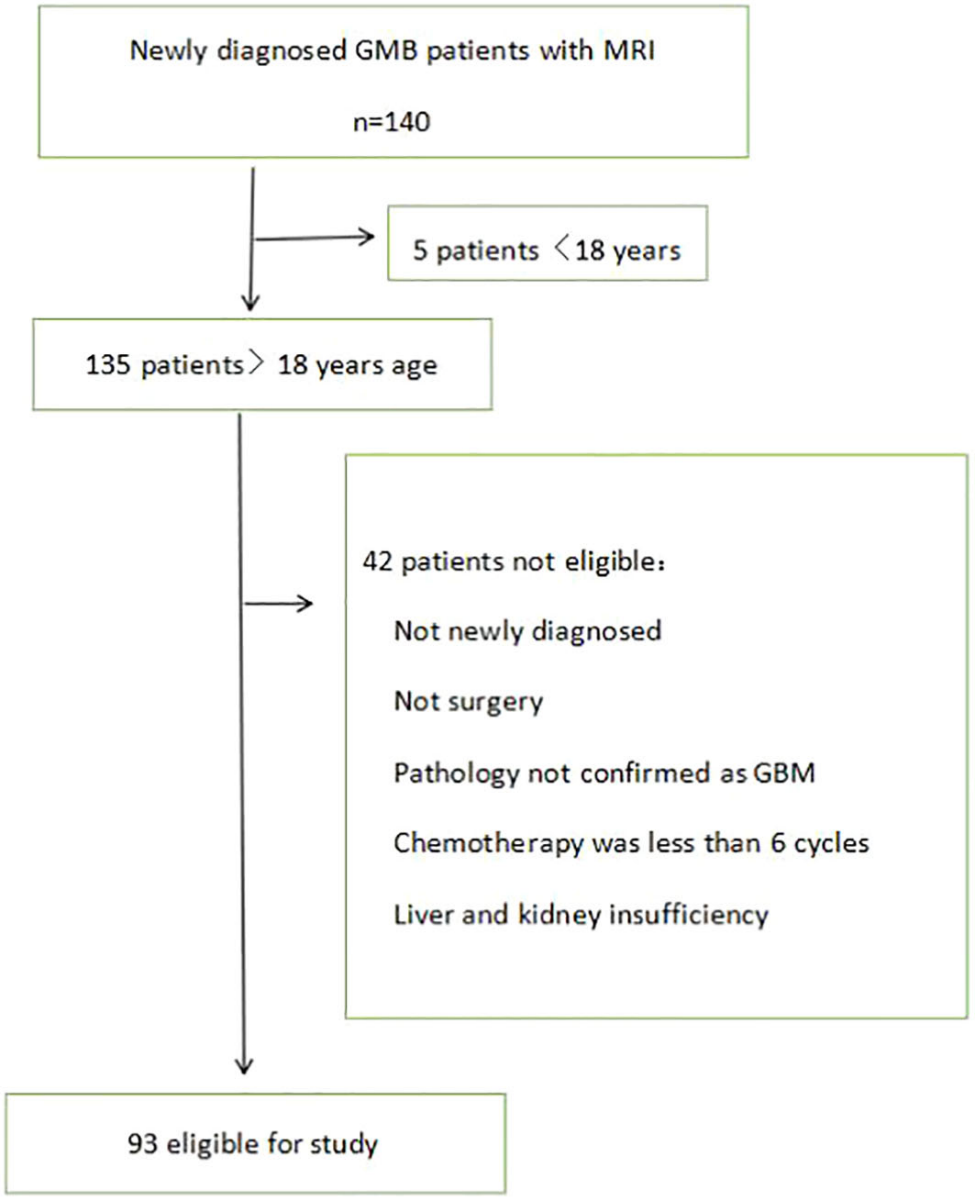
Patient identification and exclusion.

Postoperative patients with newly diagnosed GBM who hospitalized in the Affiliated Hospital of Xuzhou Medical University between January 2015 and October 2020 were recruited in this retrospective study. 140 patients with a new diagnosis of GBM were seen at hospital. Five patients were less than 18 years of age and 42 patients were not eligible for our study for the following reasons: not newly diagnosed, not surgery, pathology not confirmed as GBM, chemotherapy was less than 6 cycles, liver and kidney insufficiency (**Figure 1**).

The following clinical and pathological data of patients were collected from the electronic medical records system including age, gender, surgery method (gross total resection or subtotal resection), tumor location, tumor diameter, MRI somatotype, postoperative KPS, MGMT expression, IDH1 mutation status, p53 mutation status and expression level of Ki67.

All the enrolled patients received postoperative three dimensional conformal radiotherapy or intensity modulated radiotherapy of 60Gy within 4 weeks after surgery, TMZ at 75 mg/m^2^/day was prescribed concurrently during radiotherapy for 6 weeks, then adjuvant TMZ treatment was given at a dosage of 150 mg/m^2^/day (for the first cycle) and 200 mg/m^2^/day (for the subsequent cycles, if well tolerated) for 5 consecutive days every 28 days. Patients who received 6 cycles of adjuvant TMZ treatment with no disease progression at the end of chemotherapy were enrolled in control group and those who received more than 6 cycles were incorporated in extended group.

### Immunohistochemical staining

We considered negative MGMT expression as MGMT methylation in this study. Positive definition:the positive rate of staining ≥10%;Negative definition:the negative rate of staining <10%.Mutant IDH1 or mutant P53:the positive rate of staining ≥10% or (+);wild IDH1 and wild P53: the positive rate of staining <10% or (-).

### Follow-up

In this study, MRI was used to assess the disease progression after 6 cycles of adjuvant TMZ. According to the WHO solid tumor efficacy evaluation criteria, one or more lesions are enlarged 》25%, or appear a new lesion is the disease progression. All patients were followed up *via* a mobile phone regularly.The follow-up ended on October 31, 2021. The primary endpoints were OS and PFS.OS was calculated from the date of pathological diagnosis of GBM to the date of death of any cause or the last follow-up in surviving participants, and PFS was calculated from the date of initial surgery to the date of disease progression on MRI imaging assessed by oncologist according to the Response Assessment in Neuro-Oncology criteria or death whichever occurred first.

### Statistical analysis

Statistical analyses were performed using GraphPad Prism software (version 8.3, Inc., San Diego, CA). The association between the duration of adjuvant TMZ therapy and the clinical or pathological parameters of enrolled patients was evaluated by Chi-square test or Fisher’s exact test. OS and PFS curves were plotted by the Kaplan-Meier method and compared by the log-rank test. Univariate and multivariable analyses were compared using Cox proportional hazards model. *P* < 0.05 were considered statistically significant.

## Results

### Clinical and pathological characteristics of enrolled patients

A total of 93 postoperative patients with newly diagnosed GBM were included in this study, all the patients received complete course of radiotherapy, and their clinical and pathological characteristics were summarized in **Table 1**, 40 and 53 cases were included in control group and extended group, respectively. Among the patients in extended group, the number of patients who received 7, 8, 9, 10, 11, 12 and more than 12 cycles of adjuvant TMZ chemotherapy was 18 (33.96%), 5 (9.43%), 2 (3.77%), 5 (9.43%), 4 (7.55%), 5 (9.43%) and 14 (26.42%), respectively.

**Table 1:** Basic characteristics of enrolled postoperative patients with newly diagnosed glioblastoma.

Characteristic		Control group (*n*, %)	Extended group (*n*, %)	*P* value
**Age (years)**				0.142
	≥ 60	11 (27.50)	8 (15.09)	
	< 60	29 (72.50)	45 (84.91)	
**Gender**				0.187
	Male	21 (52.50)	35 (66.04)	
	Female	19 (47.50)	18 (33.96)	
**Surgery method**				1.000
	Gross total resection	38 (95.00)	51 (96.23)	
	Subtotal resection	2 (5.00)	2 (3.77)	
**O6-methylguanine DNA methyltransferase promoter methylation status**				0.243
	Methylated	17 (42.50)	29 (54.72)	
	Unmethylated	23 (57.50)	24 (45.28)	
**Isocitrate dehydrogenase 1 mutation status**				0.115
	Mutation	11 (27.50)	23 (43.40)	
	Wildtype	29 (72.50)	30 (56.60)	
**p53 mutation status**				0.414
	Mutation	33 (82.50)	40 (75.47)	
	Wildtype	7 (17.50)	13 (24.53)	
**Expression level of Ki67**				0.087
	≥ 35%	23 (57.50)	21 (39.62)	
	< 35%	17 (42.50)	32 (60.38)	
**Tumor location**				0.458
	Supratentorial	36 (90.00)	50 (94.34)	
	Infratentorial	4 (10.00)	3 (5.66)	
**Tumor diameter**				0.664
	< 5cm	24 (60.00)	35 (66.04)	
	> 5cm	16 (40.00)	18 (33.96)	
**MRI somatotype**				0.630
	Type I	12 (30.00)	10 (18.87)	
	Type II	7 (17.50)	9 (16.98)	
	Type III	9 (22.50)	15 (28.30)	
	Type IV	12 (30.00)	19 (35.85)	
**Postoperative KPS**				0.292
	< 80	20 (50.00)	20 (37.74)	
	> 80	20 (50.00)	33 (62.26)	

In order to avoid bias influencing the results of analysis, we used receiver operating characteristic curve to calculate the optimal cut-off value of Ki67, and the results indicated that the optimal cut-off value of Ki67 was 35%. Finally, there was 44 patients with higher expression of Ki67 (≥ 35%, 23 cases and 21 cases in control group and extended group, respectively) and 49 patients with lower expression of Ki67 (< 35%, 17 cases in control group and 32 cases in extended group).

### Extended adjuvant TMZ chemotherapy significantly prolonged OS and PFS of patients with newly diagnosed GBM

**Figure 2:**
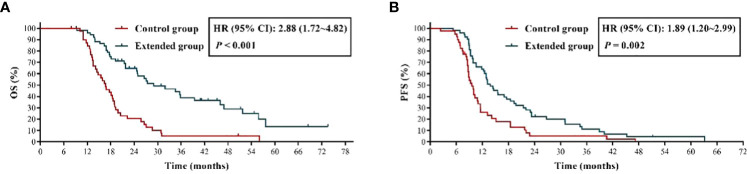
The effects of extended adjuvant temozolomide chemotherapy on the clinical outcomes of patients with newly diagnosed glioblastoma. **(A)** The Kaplan−Meier survival curves for overall survival. **(B)** The Kaplan−Meier survival curves for progression-free survival.

We firstly evaluated the effect of extended adjuvant TMZ chemotherapy on the OS and PFS of these patients, the results (**Figure 2A**) showed that the OS of patients received more than 6 cycles of TMZ treatment was significant longer than those received 6 cycles of TMZ chemotherapy [mOS: 29.00 months *vs.* 16.70 months; *P* < 0.001; hazard ratio (HR) = 2.88, 95% confidence interval (CI): 1.72 ~ 4.82]. Additionally, we observed a shorter mPFS in control group than extended group (9.60 months *vs.* 13.80 months, HR = 1.89, 95% CI: 1.20 ~ 2.99, *P* = 0.002) (**Figure 2B**).

### Newly diagnosed GBM patients with MGMT promoter methylation benefited the most from extended adjuvant TMZ chemotherapy

**Figure 3:**
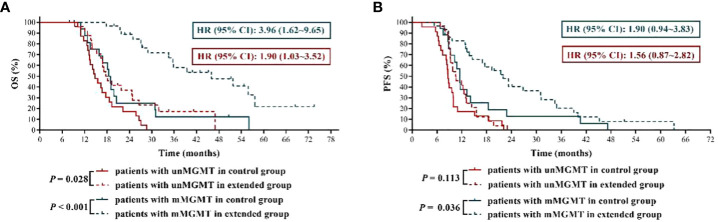
The effects of extended adjuvant temozolomide chemotherapy on the clinical outcomes of newly diagnosed glioblastoma patients with different O6-methylguanine DNA methyltransferase promoter methylation statuses. **(A)** The Kaplan−Meier survival curves for overall survival. **(B)** The Kaplan−Meier survival curves for progression-free survival.

It is generally believed that GBM patients with MGMT promoter methylation have a better response to TMZ treatment (11), therefore, we inferred that the application of extended TMZ chemotherapy in the patients with MGMT promoter methylation might have more far-reaching value. The results (**Figure 3**) showed that patients with methylated MGMT promoter in extended group had significantly longer OS and PFS than those in control group (mOS: 46.00 months *vs.* 18.70 months, HR = 3.96, 95% CI: 1.62 ~ 9.65, *P* < 0.001; mPFS: 22.00 months *vs.* 11.50 months, HR = 1.90, 95% CI: 0.94 ~ 3.83, *P* = 0.036). Moreover, compared with 6 cycles of TMZ treatment, although extended TMZ chemotherapy did not obviously improve the PFS in newly diagnosed GBM patients with unmethylated MGMT promoter (mPFS: 10.55 months *vs.* 8.70 months, HR = 1.56, 95% CI: 0.87 ~ 2.82, *P* = 0.113), their OS was remarkably prolonged (mOS: 17.85 months *vs.* 14.80 months, HR = 1.90, 95% CI: 1.03 ~ 3.52, *P* = 0.028) (**Figure 3**). To sum up, newly diagnosed GBM patients with methylated MGMT promoter benefited the most from extended adjuvant TMZ treatment.

### Newly diagnosed GBM patients with IDH1 mutation were more suitable for extended adjuvant TMZ chemotherapy

**Figure 4:**
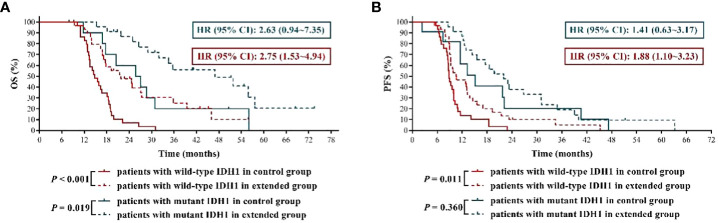
The effects of extended adjuvant temozolomide chemotherapy on the clinical outcomes of newly diagnosed glioblastoma patients with different isocitrate dehydrogenase 1 mutation statuses. **(A)** The Kaplan−Meier survival curves for overall survival. **(B)** The Kaplan−Meier survival curves for progression-free survival.

Previous studies had demonstrated that IDH1 mutation was associated with favorable clinical outcomes in GBM patients (12, 13), which was further validated in this study by the results that GBM patients with mutant IDH1 had significantly longer OS and PFS than those with wild-type IDH1 in control group (mOS: 26.45 months *vs.* 14.80 months, HR = 2.85, 95% CI: 1.51 ~ 5.39, *P* = 0.002; mPFS: 15.10 months *vs.* 8.80 months, HR = 2.46, 95% CI: 1.31 ~ 4.61, *P* = 0.005) (**Figure 4**). More importantly, we reconfirmed that, compared with IDH1 wild-type GBM patients, IDH1 mutant GBM patients were more sensitive to TMZ chemotherapy (12), because our results (**Figure 4**) showed that the mOS and mPFS of IDH1 mutant GBM patients in extended group were longer by about 20 months and 7 months, respectively, than those in control group (mOS: 46.90 months *vs.* 26.45 months, HR = 2.63, 95% CI: 0.94 ~ 7.35, *P* = 0.019; mPFS: 22.00 months *vs.* 15.10 months, HR = 1.41, 95% CI: 0.63 ~ 3.17, *P* = 0.360), while the disparities in mOS and mPFS of IDH1 wild-type GBM patients between the two groups were 7 months and less than 2 months, respectively (mOS: 21.80 months *vs.* 14.80 months, HR = 2.75, 95% CI: 1.53 ~ 4.94, *P* < 0.001; mPFS: 10.55 months *vs.* 8.80 months, HR = 1.88, 95% CI: 1.10 ~ 3.23, *P* = 0.011). The above results indicated that newly diagnosed GBM patients with IDH1 mutation were more suitable for extended adjuvant TMZ chemotherapy.

### Newly diagnosed GBM patients could benefit to some extent from extended adjuvant TMZ chemotherapy regardless of p53 mutation status

**Figure 5:**
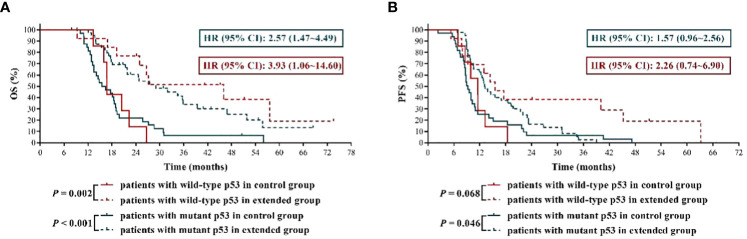
The effects of extended adjuvant temozolomide chemotherapy on the clinical outcomes of newly diagnosed glioblastoma patients with different p53 mutation statuses. **(A)** The Kaplan−Meier survival curves for overall survival. **(B)** The Kaplan−Meier survival curves for progression-free survival.

At present, the effect of p53 mutation status on TMZ sensitivity of GBM remains to be elusive. In this study, we found that, compared with p53 wild-type GBM patients who received TMZ treatment, the mOS and mPFS of p53 mutant GBM patients were shortened to some extent, although the differences were not all statistically significant (mOS of control group: 15.95 months *vs.*16.80 months, *P* = 0.949; mOS of extended group: 29.00 months *vs.*46.00 months, *P* = 0.309; mPFS of control group: 9.30 months *vs.*11.50 months, *P* = 0.940; mPFS of extended group: 13.15 months *vs.*15.50 months, *P* = 0.039). Then we analyzed whether there was significant difference in the prognostic effect of extended TMZ chemotherapy on GBM population with different p53 mutation status. The results shown in **Figure 5A** indicated that, compared with 6 cycles of TMZ chemotherapy, more than 6 cycles of TMZ resulted in more than 2 times and about 1.8 times longer mOS in GBM patients with wild-type p53 and mutant p53, respectively (p53 wild-type GBM patients: 46.90 months *vs.* 16.80 months, HR = 3.93, 95% CI: 1.06 ~ 14.60, *P* = 0.002; p53 mutant GBM patients: 29.00 months *vs.* 15.95 months, HR = 2.57, 95% CI:1.47 ~ 4.49, *P* < 0.001). Furthermore, while extended TMZ treatment did not significantly prolong PFS in p53 wild-type GBM patients (mPFS: 15.50 months *vs.* 11.50 months, HR = 2.26, 95% CI: 0.74 ~ 6.90, *P* = 0.068), this treatment regimen did dramatically improve PFS in GBM patients with mutant p53 (mPFS: 13.15 months *vs.* 9.30 months, HR = 1.57, 95% CI: 0.96 ~ 2.56, *P* = 0.046) (**Figure 5B**).

### Extended adjuvant TMZ treatment dramatically improved the OS and PFS of newly diagnosed GBM patients with higher expression level of Ki67

**Figure 6:**
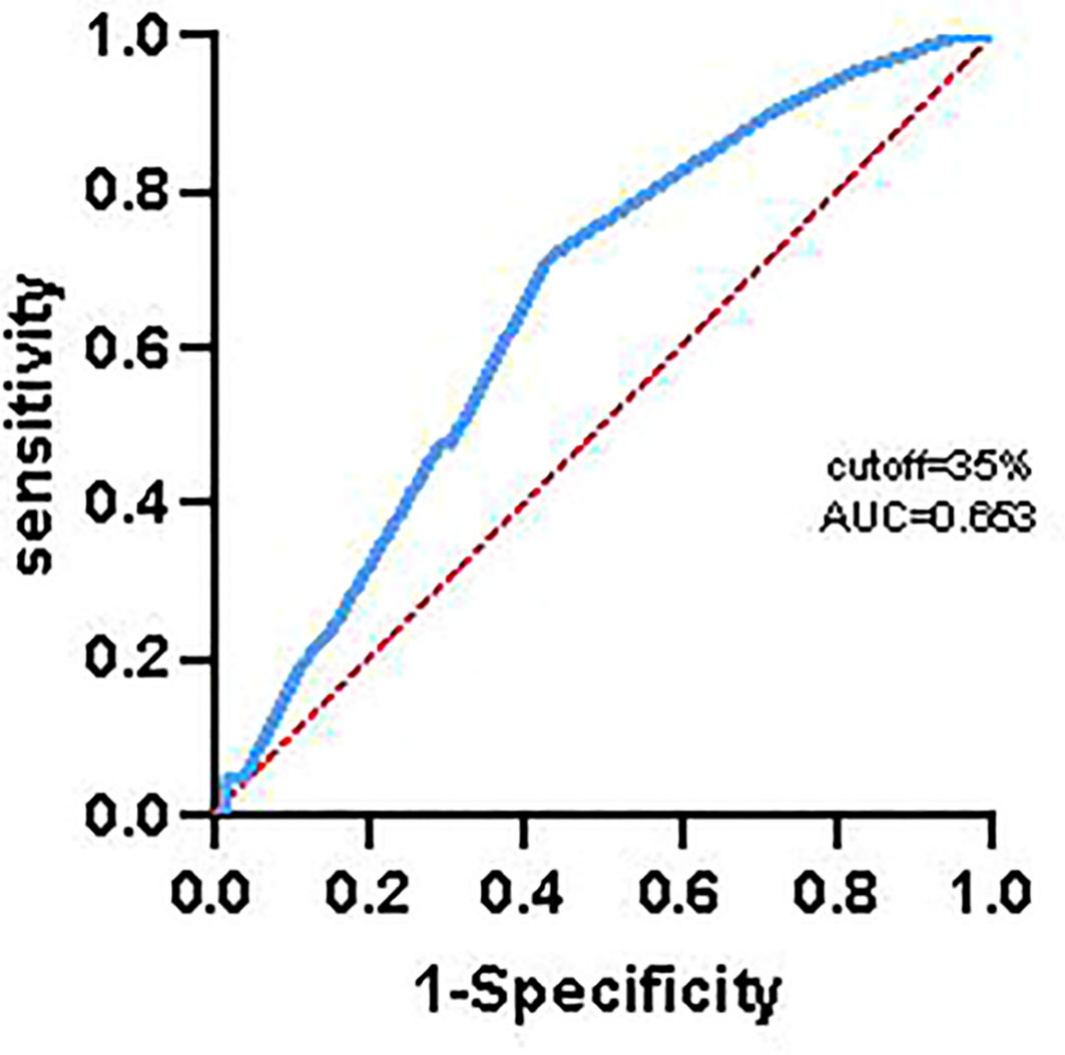
Ki67 of receiver operating characteristic curve.

**Figure 7:**
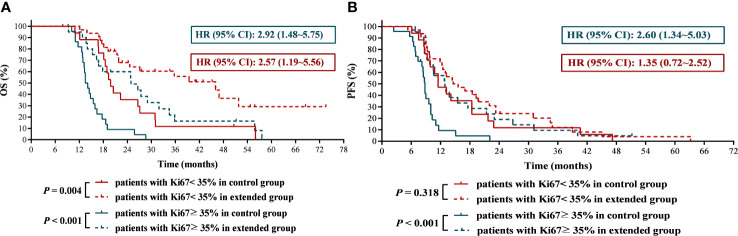
The effects of extended adjuvant temozolomide chemotherapy on the clinical outcomes of newly diagnosed glioblastoma patients with different expression level of Ki67. **(A)** The Kaplan−Meier survival curves for overall survival. **(B)** The Kaplan−Meier survival curves for progression-free survival.

Some studies have shown that the expression of Ki67 is related to chemotherapy sensitivity in breast cancer and rectal adenocarcinoma (14, 15). In this study, we obtained a cutoff value of Ki67 of 35% by receiver operating characteristic curve (**Figure 6**).The results found that the mOS and mPFS of patients with lower Ki67 expression who received 6 cycles of TMZ treatment was significantly longer than those with higher Ki67 expression (mOS: 19.80 months *vs.* 13.80 months, HR = 0.36, 95% CI: 0.18 ~ 0.70, *P* < 0.001; mPFS: 11.50 months *vs.* 8.80 months, HR = 0.43, 95% CI:0.22 ~ 0.82, *P* = 0.004), which suggested that GBM patients with lower Ki67 expression were more sensitive to TMZ chemotherapy than those with higher expression of Ki67. Significantly, compared with 6 cycles of TMZ chemotherapy, extended TMZ treatment obviously prolonged the OS of newly diagnosed GBM patients regardless of Ki67 expression levels (patients with Ki67 < 35%: 19.80 months *vs.* 46.00 months, HR = 2.57, 95% CI: 1.19 ~ 5.56, *P* = 0.004; patients with Ki67 ≥ 35%: 13.80 months *vs.* 24.90 months, HR = 2.92, 95% CI: 1.48 ~ 5.75, *P* < 0.001) (**Figure 7A**). However, although the strategy of extended TMZ treatment dramatically improved the PFS of newly diagnosed GBM patients with higher expression of Ki67 (8.80 months *vs.* 12.90 months, HR = 2.60, 95% CI: 1.34 ~ 5.03, *P* < 0.001), it did not significantly improve the PFS in the population with lower Ki67 expression (11.50 months *vs.* 15.10 months, HR = 1.35, 95% CI: 0.72 ~ 2.52, *P* = 0.318) (**Figure 7B**).

### Adverse events

Neutropenia was the most frequently observed treatment related hematologic adverse events (n=28, 30.1%). The most frequent nonhematologic adverse events were nausea and Vomiting (n=46, 49.5%) (**Table 2**). There was no statistically significant difference in the toxicity profile between six-cycle group and > six-cycle group (**Table 2**).There was no statistically significant difference in the rate of grade 3 & 4 treatment related toxicities between both groups (**Table 3**). No patient delayed chemotherapy or changed the dose of chemotherapeutic drugs due to adverse effects.

**Table 2:** Comparison of the toxicities between six-cycle treated patients and > six-cycle treated patients.

Adverse events	6 cycles (n=40)	>6 cycles (n=53)	P value
Hematologic			
Leukopenia	6 (15.00)	12 (22.64)	0.356
Neutropenia	12 (30.00)	16 (30.19)	0.984
Anemia	3 (7.50)	7 (13.21)	0.379
Thrombocytopenia	8 (20.00)	15 (28.30)	0.358
Fatigue	12 (30.00)	23 (43.40)	0.187
Nausea、Vomiting	20 (50.00)	26 (49.06)	0.928
Constipation	4 (10.00)	8 (15.09)	0.468
Pneumonia	3 (7.50)	5 (9.43)	0.523
hepatotoxicity	0	3 (5.66)	0.257

**Table 3:** Grade 3 & 4 CTC that occurred during adjuvant course of TMZ.

Adverse events	6 cycles (n=40)	>6 cycles (n=53)	P value
Hematologic			
Leukopenia	2 (7.50)	3 (9.43)	1.00
Neutropenia	1 (2.50)	3 (5.66)	0.632
Anemia	0	0	
Thrombocytopenia	0	1 (1.89)	0.257
Fatigue	0	0	
Nausea、Vomiting	0	0	
Constipation	0	0	
Pneumonia	0	0	
hepatotoxicity	0	0	

Association of age, gender, surgery method, MGMT promoter methylation status, IDH mutation status, P53 mutation status, expression level of Ki67, tumor location, tumor diameter, MRI somatotype, postoperative KPS and TMZ cycles with outcome.

In this study, we analyzed MRI features of GBMs in specific relation to the SVZ. Classification was as follows by preoperative MR images:group I, CEL contacting SVZ and infiltrating cortex; group II, CEL contacting SVZ but not involving cortex; group III, CEL not contacting SVZ but involving cortex; and group IV, CEL neither contacting SVZ nor infiltrating cortex (16).

Univariate analysis using the Cox proportional hazard model was performed to assess their association with PFS or OS. Surgery method, MGMT promoter methylation status, IDH mutation status, expression level of Ki67, tumor location, MRI somatotype, postoperative KPS and TMZ cycles were favorable factors for overall survival. In addition, MGMT promoter methylation status, IDH mutation status, expression level of Ki67, tumor location, MRI somatotype, postoperative KPS and TMZ cycles were favorable factors for progression-free survival. Multivariate analysis using the Cox proportional hazard model to reveal MGMT promoter methylation status, IDH mutation status, expression level of Ki67, tumor location, MRI somatotype, postoperative KPS and TMZ cycles to be strongly associated with OS. In addition, multivariate analysis demonstrated MGMT promoter methylation status, IDH mutation status, MRI somatotype and TMZ cycles to be associated with PFS (**Tables 4**, **5**).

**Table 4:** Univariate and multivariate Cox proportional hazards model results for overall survival.

Factors	Univariate COX	*P*	Multivariate COX	P
	*HR (95%CI)*		*HR (95%CI)*	
Age	1.332 (0.747, 2.376)	0.332	–	–
Gender	1.213 (0.744, 1.978)	0.439	–	–
Extent of resection	7.802 (2.259,26.945)	0.001	2.661 (0.736,9.616)	0.136
promoter methylation status	2.803 (1.691, 4.648)	<0.001	2.150 (1.173,3.939)	0.013
IDH1 mutation state	3.312 (1.878, 5.842)	<0.001	2.719 (1.471,5.024)	0.001
P53 mutation state	1.168 (0.647, 2.108)	0.607	–	–
Ki67 expression	2.429 (1.488, 3.966)	<0.001	2.009 (1.126, 3.586)	0.018
Chemotherapy cycle	4.184 (2.526, 6.930)	<0.001	7.707 (4.101, 14.484)	<0.001
Tumor location	2.867 (1.296,6.344)	0.009	1.073 (0.430,2.682)	0.879
Tumor diameter	1.215 (0.732,2.019)	0.451	–	–
MRI somatotype		<0.001		<0.001
	0.413 (0.196,0.872)	0.020	0.353 (0.160,0.779)	0.010
	0.216 (0.107,0.436)	<0.001	0.179 (0.078,0.411)	<0.001
	0.202 (0.103,0.395)	<0.001	0.150 (0.066,0.341)	<0.001
Postoperative KPS	5.815 (3.144,10.755)	<0.001	5.090 (2.531,10.234)	<0.001

**Table 5:** Univariate and multivariate Cox proportional hazards model results for progression-free survival.

Factors	Univariate COX	P	Multivariate COX	P
	HR (95%CI)		HR (95%CI)	
Age	1.348 (0.807,2.252)	0.253	–	–
Gender	1.049 (0.681,1.618)	0.828	–	–
Extent of resection	2.112 (0.646,6.906)	0.216	–	
promoter methylation status	3.138 (1.952,5.046)	<0.001	2.842 (1.644,4.913)	<0.001
IDH1 mutation state	3.029 (1.893,4.845)	<0.001	3.426 (1.981,5.925)	<0.001
P53 mutation state	1.500 (0.880,2.559)	0.136	–	–
Ki67 expression	1.750 (1.137,2.694)	0.011	1.266 (0.783,2。049)	0.336
				
Chemotherapy cycle	2.580 (1.654,4.023)	<0.001	3.290 (1.984,5.455)	<0.001
Tumor location	2.403 (1.082, 5.335)	0.031	1.272 (0.541, 2.989)	0.581
Tumor diameter	0.944 (0.601, 1.482)	0.801	–	–
MRI somatotype		0.001		0.030
	0.445 (0.222, 0.889)	0.22	0.519 (0.259, 1.037)	0.063
	0.351 (0.186, 0.664)	0.001	0.507 (0.257, 0.997)	0.049
	0.279 (0.149, 0.521)	〈0.001	0.357 (0.181, 0.705)	0.003
Postoperative KPS	2.708 (1.698, 4.318)	<0.001	1.761 (0.999, 3.103)	0.050

## Discussion

The clinical outcomes of newly diagnosed GBM patients who receive STUPP regimen remains dismal, because approximately 70% of patients progress within one year of the ending of treatment (17). Hence, it is strongly necessary to modify the therapeutic strategy in order to improve the prognosis of these patients. The impact of extended TMZ therapy in newly diagnosed GBM remains a topic of discussion.

In adult GBM patients with prolonged TMZ adjuvant chemotherapy, the risk of tumor recurrence in the long-cycle group was significantly lower than that in the standard 6-cycle treatment group, extended maintenance TMZ treatment improved progression-free survival (p=0.03) and overall survival (p=0.001) (18). GIUSEPPE et al (19), this study conducted 37 patients who underwent operations for glioblastoma. Kaplan-Meier curve analysis showed that patients treated with more than 6 TMZ cycles had OS and PFS that was significantly longer than patients receiving standard treatment (median OS 28 months vs 8 months, respectively; p = 0.0001; median PFS 20 months vs 4 months, respectively; p =0.0002). The extended TMZ regimen was associated with a nonsignificant improvement in PFS without corresponding improvement in OS (20).The findings are inconsistent with this study stating that extended TMZ treatment was associated with increased OS (mOS: 29.00 months vs. 16.70 months; P < 0.001; HR = 2.88, 95% CI: 1.72 ~ 4.82)and PFS (mPFS:9.60 months vs. 13.80 months; P = 0.002; HR = 1.89, 95% CI: 1.20 ~ 2.99).However, the finding might be affected by the retrospective record and ethnic difference.

Multiple studies have revealed that MGMT plays a vitally important role in the mechanism of resistance to alkylating agents such as TMZ, and cancers with low level of MGMT expression are more tend to have a better response to TMZ (21–23). Hence, dose-dense TMZ regimens including a continuous daily schedule at a dose of 50 mg/m^2^/d (24), the 7 of 14-day schedule at a dose of 150 mg/m^2^/d (25) and the 21 of 28-day schedule at a dose of 75 to 100 mg/m^2^/d (26) were proposed with the idea that they could potentially reduce MGMT level in cancer cells by overwhelming the cells’ ability to synthesize MGMT, and thus might improve TMZ’s therapeutic activity (27). In order to detect the hypothesis that prolonged exposure to TMZ improves prognosis in patients with newly diagnosed GBM, a randomized phase III clinical trial was initiated, but it is regrettable that the study did not demonstrate improved efficacy for dose-dense TMZ in newly diagnosed GBM patients, regardless of MGMT methylation status, and there was increased grade ≥ 3 toxicity in dose-dense TMZ group (8). However, another study found that dose-dense regimen for newly diagnosed GBM appeared promising with 1-year survival rate of 80% (28). Recently, a phase II randomized, multicenter, open-label trial conducted by Carmen Balana et al. revealed that extending adjuvant TMZ for more than 6 cycles leaded to greater toxicity but conferred no additional benefit in PFS and OS (10). These results mean that the optimal dose-dense TMZ regimen for newly diagnosed GBM still is a subject of debate. In this study, we found that newly diagnosed GBM patients with negative MGMT benefited significantly from extended adjuvant TMZ treatment with obviously prolonged OS and PFS, which provided supporting evidence for the application of extended adjuvant TMZ therapy in newly diagnosed GBM patients with negative MGMT. Although MGMT methylation was not tested by molecular detection in this study, the level of MGMT expression can still reflect the sensitivity of glioma to temozolomide chemotherapy (21). Glioblastoma with MGMT negative results had a better prognosis.

Some studies demonstrated that GBM patients with IDH1 mutation showed a better response to radiotherapy and TMZ chemotherapy (12, 29, 30). Consistent with these studies, we further validated that newly diagnosed GBM patients with mutant IDH1 possessed a significantly better OS and PFS than those with wild-type IDH1. The somatic genomic landscape of GBM showed that deregulate p53 was found in 85% ~ 90% of GBM, including 27.9% of p53 gene mutations or deletions (31). However, the effect of p53 mutation on TMZ sensitivity in GBM is not uniform. In this study, we found that there were no significant difference for OS and PFS between wild-type p53 GBM patients and mutant p53 GBM patients who received STUPP regimen, and whether GBM patients benefit from extended TMZ chemotherapy was independent of p53 mutation status, this may be related to the complicated p53 signaling pathway. As a nuclear antigen for cell proliferation, Ki67 antigen is mainly used to evaluate the proliferative activity of tumor cells. In the study of Zheng Y et al. they evidenced that programmed cell death-ligand 1 containing exosomes promoted cancer growth and Ki67 protein expression to increase the resistance to TMZ in GBM (32), which implied that Ki67 was involved in the development of TMZ resistance. Similarly, our results suggested that GBM patients with higher expression of Ki67 suffered remarkably worse OS and PFS than those with lower expression of Ki67, and except for the patients with lower Ki67 expression, extended TMZ chemotherapy also significantly benefited GBM patients with higher Ki67 expression with prolonged OS and PFS.

A study demonstrates that GBMs with both SVZ and cortical involvement and SVZ contact alone result in shorter progression-free survival and overall survival. Thus, SVZ involvement may serve as a prognostic indicator (33). This is consistent with the findings of this study. At the same time, multivariate analysis also confirmed MGMT promoter methylation status, IDH mutation status, expression level of Ki67, tumor location, postoperative KPS and TMZ cycles to be strongly associated with overall survival, and MGMT promoter methylation status, IDH mutation status, postoperative KPS and TMZ cycles were independent prognostic factors for progression-free survival.

In our study, the overall toxicity profile of adjuvant TMZ therapy was tolerable as most of the patients (n=45, 81.8%) developed only grade 1 or 2 adverse events. Therefore, long-cycle TMZ chemotherapy did not show significant toxic side effects, it was safe and reliable.

We acknowledge several potential limitations of this study. First, this was a nonrandomized retrospective and single-center study, and the selection bias was inevitable. Second, the sample size was relatively small. Third, the number of chemotherapy cycles of TMZ in the extended group was not consistent, which might affect the analysis results to a certain extent. Forth, This study used Immunohistochemical examination without molecular detection. So the findings of this study need to be further validated in additional larger, multicenter, and prospective clinical trials.

## Conclusion

This study provided more evidence to support the view that the therapeutic schedule of extended adjuvant TMZ significantly prolonged OS and PFS of patients with newly diagnosed GBM regardless of p53 mutation status, and patients with IDH1 mutation were more suitable for this treatment. Compared with MGMT unmethylated GBM patients, newly diagnosed GBM patients with MGMT promoter methylation benefited the most from extended adjuvant TMZ chemotherapy. Moreover, extended adjuvant TMZ treatment dramatically improved the OS and PFS of newly diagnosed GBM patients with higher expression level of Ki67.

## Data availability statement

The original contributions presented in the study are included in the article/supplementary material. Further inquiries can be directed to the corresponding authors.

## Author contributions

LZ and XD designed the research; TW, WL, NZ, and XC collected data; JC, HQ, and TW analyzed the results and wrote the manuscript. All authors critically reviewed the final draft prior to submission.

## Funding

This work was supported by the National Natural Science Foundation of China (No.81972845) and the Introduction of Specialist Team in Clinical Medicine of Xuzhou (2019TD003).

## Conflict of interest

The authors declare that the research was conducted in the absence of any commercial or financial relationships that could be construed as a potential conflict of interest.

## Publisher’s note

All claims expressed in this article are solely those of the authors and do not necessarily represent those of their affiliated organizations, or those of the publisher, the editors and the reviewers. Any product that may be evaluated in this article, or claim that may be made by its manufacturer, is not guaranteed or endorsed by the publisher.
